# Supplementary material for: Finding differentially expressed sRNA-Seq regions with srnadiff
Source: PLoS One. 2021 Aug 20;16(8):e0256196. doi: 10.1371/journal.pone.0256196 (PMC8378736; doi:10.1371/journal.pone.0256196)
Supplement: S1 Appendix — Supplementary figures, other benchmarking, code used, tool versions, and DOI of the preprocessed data are given in the Additional Data file. (PDF) [file pone.0256196.s001.pdf]

Supporting Information
Finding differentially expressed sRNA-Seq regions with srnadiff

Additional figures

Human dataset

DESeq2 quality control in the HMM step

Figure 1 shows two quality control plots.

Figure 1a shows the dispersion of the counts, compared to the mean value. The plot shows that the dispersion accumulates near the fitted distribution, which is what we expect.

Figure 1b shows the distribution of p-values. These distribution can be decomposed into a flat distribution (the null hypothesis), and a peak on 0 (the alternative hypothesis). The small peak at 1 contains all the regions which have been detected as outliers by DESeq2, and not considered for differential expression testing.

Region size distribution

We plotted the distribution of the sizes of the regions predicted by each tool (see Figure 2). We noted that srnadiff and derfinder could preferentially retrieve short regions, consisting primarily of short loci such as miRNAs, tRFs, etc. However, ShortStack can capture longer regions, which is a clear advantage for this method.

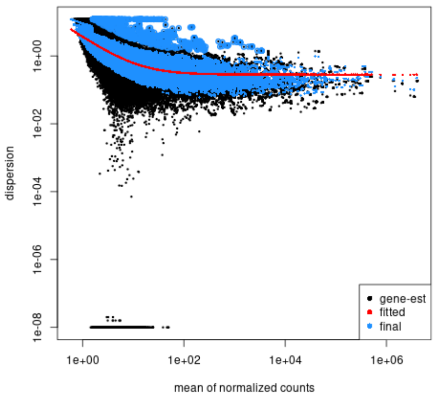

(a) Dispersion of the counts.

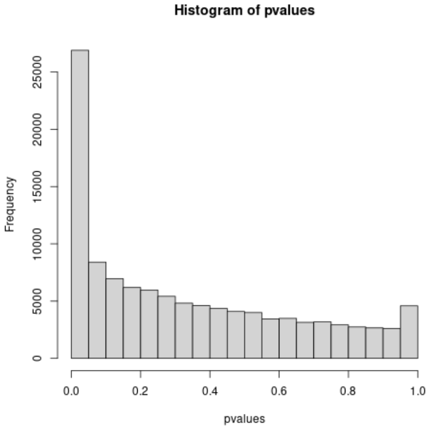

(b) p-values of the counts.

Figure 1: **Results given by DESeq2, during the HMM step.**

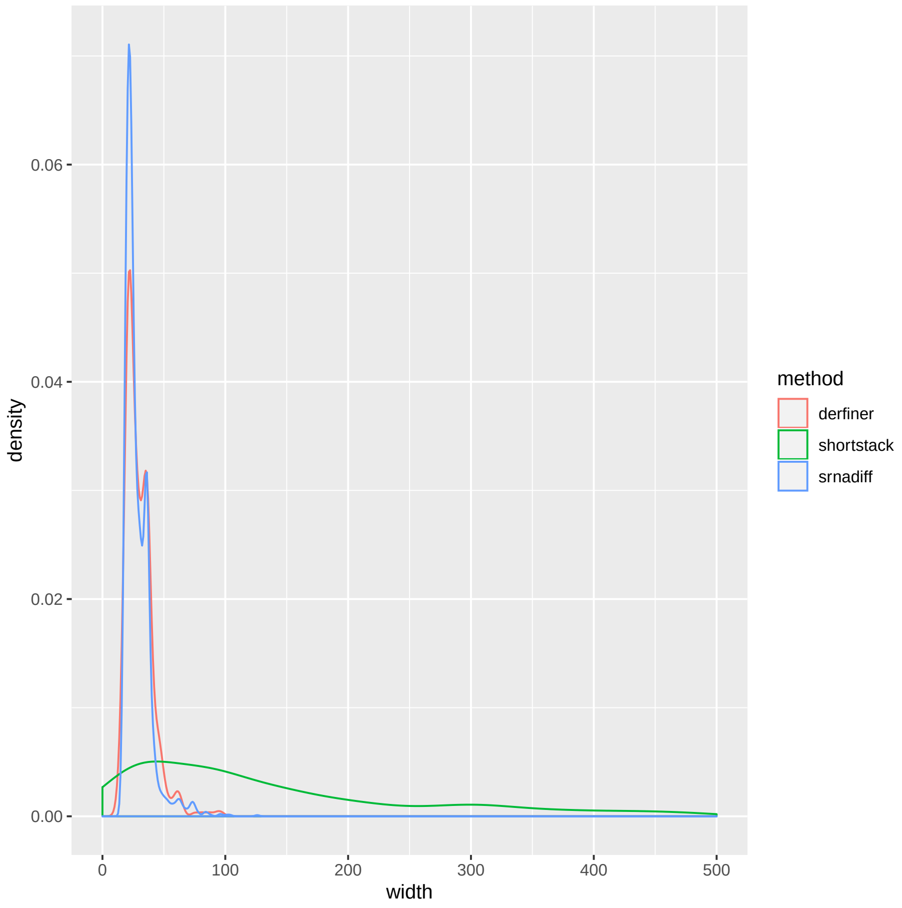

Figure 2: **Region size distributions.**

Pairwise size distribution

Here, we first retrieved all the regions found by derfinder and ShortStack, that overlapped with a region found by srnadiff, on at least 80% of the region found by srnadiff, or on at least 80% of the region found by the other tool. We then compared the sizes of the overlapping regions on Figure 3, where the x-axis gives the size of the region found by srnadiff, and on the y-axis, the size of the region found by the other tool. We can note that srnadiff and derfinder give regions with comparable similar sizes. However, ShortStack usually produces much longer regions.

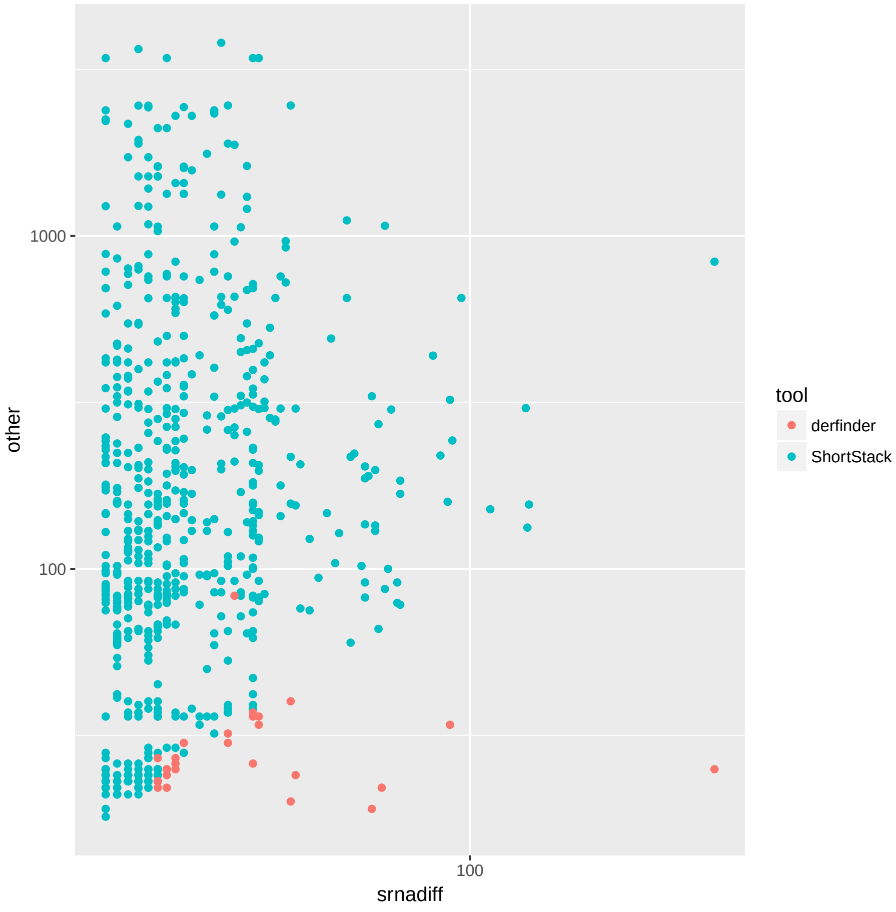

Figure 3: **Pairwise region size distribution.**

Number of features per region

We wondered whether ShortStack, since it produces longer regions, could potentially "merge" several differentially expressed sRNAs into a unique region. So, we computed the number of features per region in Figure 4. It seems that ShortStack merges more regions than the other tools (~25% *vs* ~15% for the other tools).

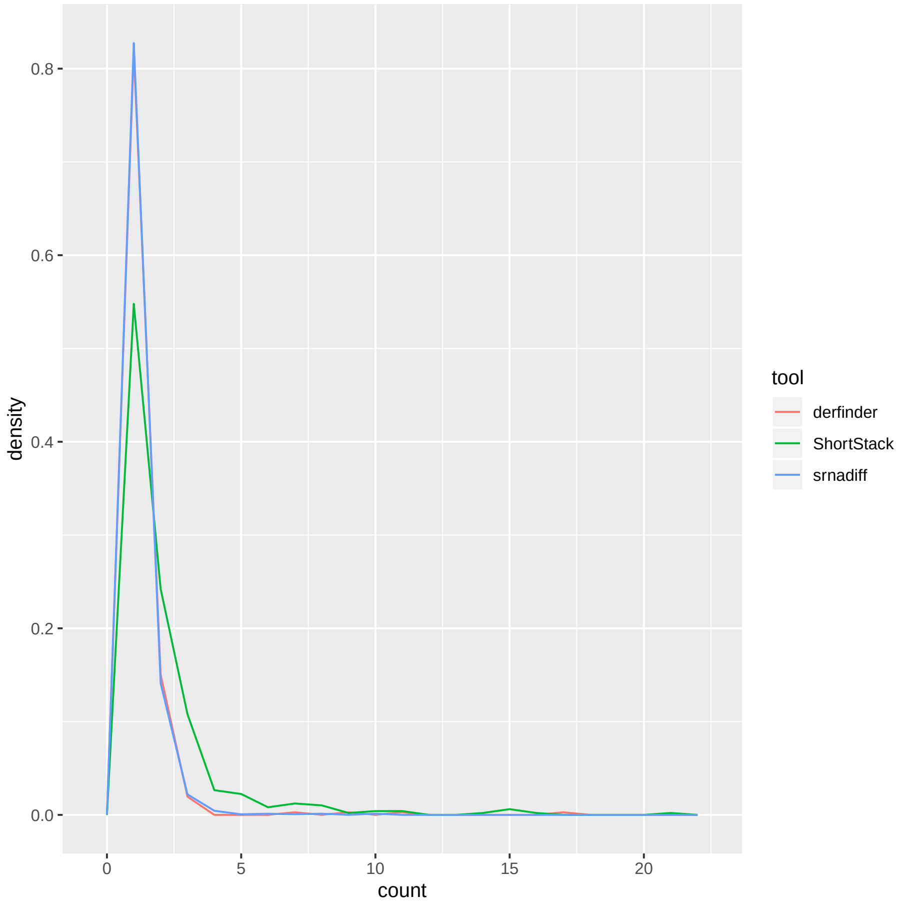

Figure 4: **Number of overlapped annotation per region.**

Pairwise p-value comparisons

We then took all the regions provided by derfinder and ShortStack that where 80% identical with the regions found by srnadiff, and compared their p-values (see Figure 5). We expected them to be nearly identical, but it seems that derfinder gives lower p-values, when the regions are comparable.

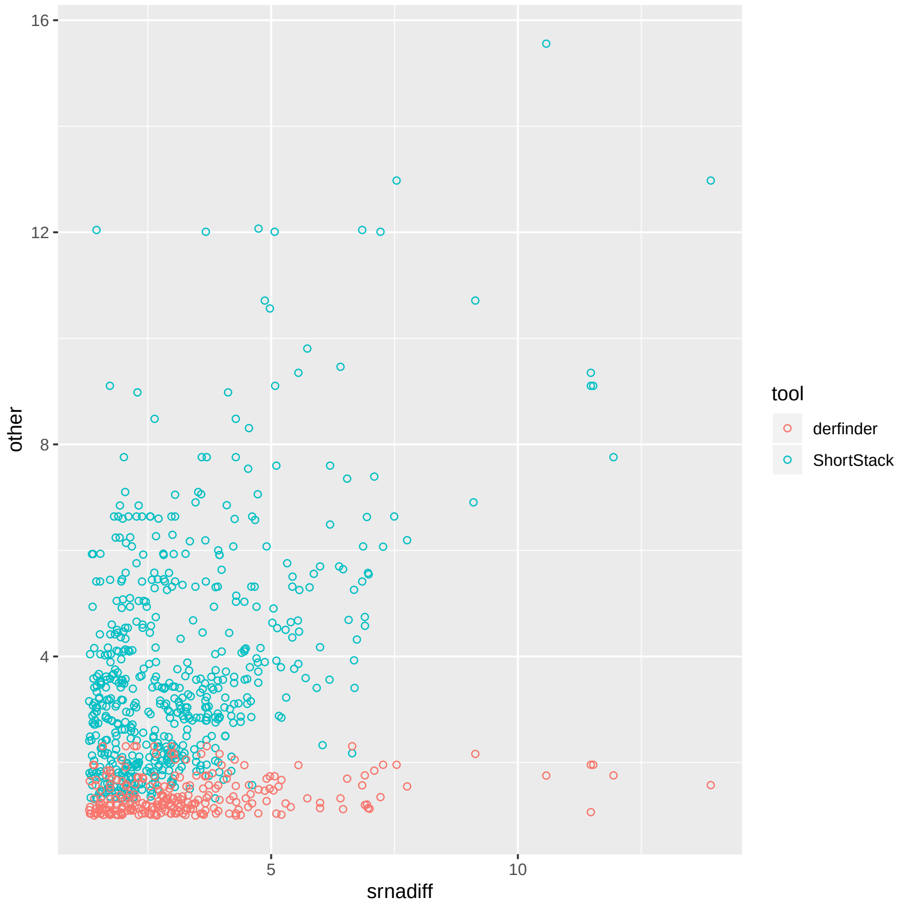

Figure 5: **Pairwise adjusted p-value comparisons.** The distribution of the $-\log_{10}$ of these values are plotted.

Differentially expressed regions

We used IGV to show the distribution of the reads on two differentially expressed regions, uniquely found by srnadiff (see Figure 6).

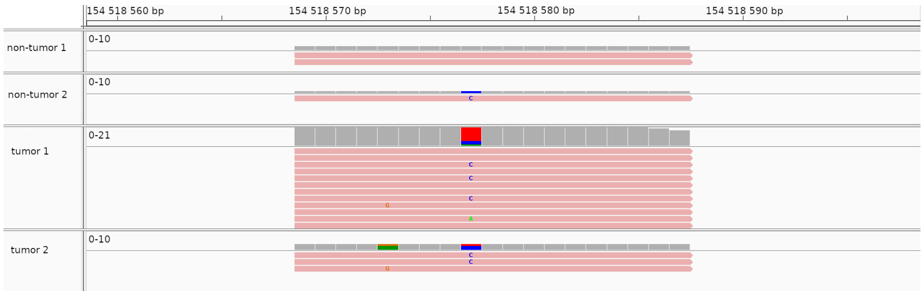

(a) Region is 2:154518571–154518588, and reads are 19bp long.

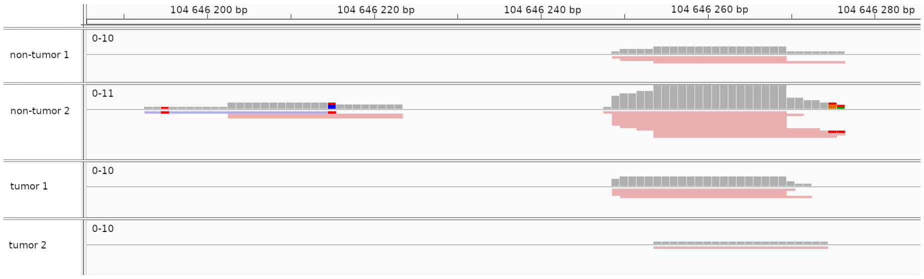

(b) Region is 6:104646205–104646269, and reads are 21bp long.

Figure 6: **Screenshots of differentially expressed regions found by srnadiff.** The two first tracks are non-tumor cells, whereas the two bottom tracks are tumor cells. Each track is divided into sub-tracks: the top sub-track shows the coverage (the scale is in the top left corner); the bottom sub-track shows the read distribution. In both examples, the region is more expressed in non-tumor cells. Mutation with respect to the assembled genome are show in red when the sequenced nucleotide is T, and blue of C. Reads of the second plot seem to be edited at their 3' ends.

A. thaliana dataset

Region size distribution

Here again, ShortStack tends to give longer regions, and miss shorter ones (see Figure 7).

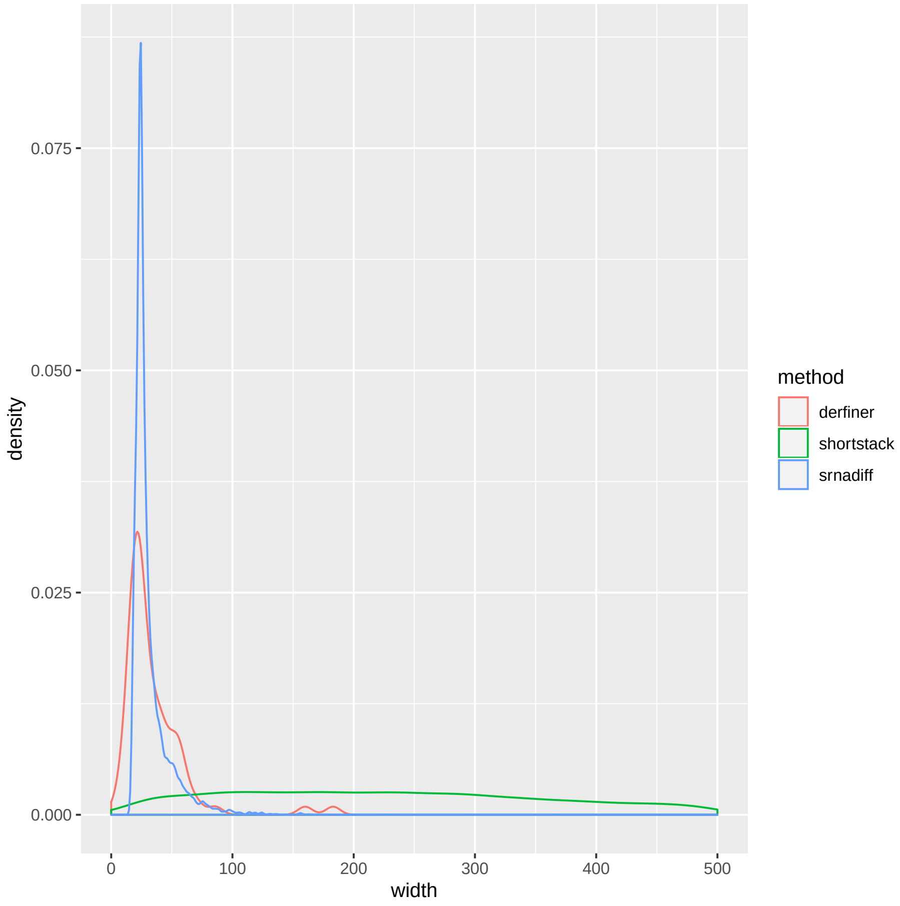

Figure 7: **Region size distributions.**

Pairwise size distribution

The pairwise comparison of the size distribution confirms that ShortStack find longer regions (see Figure 8). Even when the regions found by srnadiff and ShortStack overlap, ShortShack will produce longer regions.

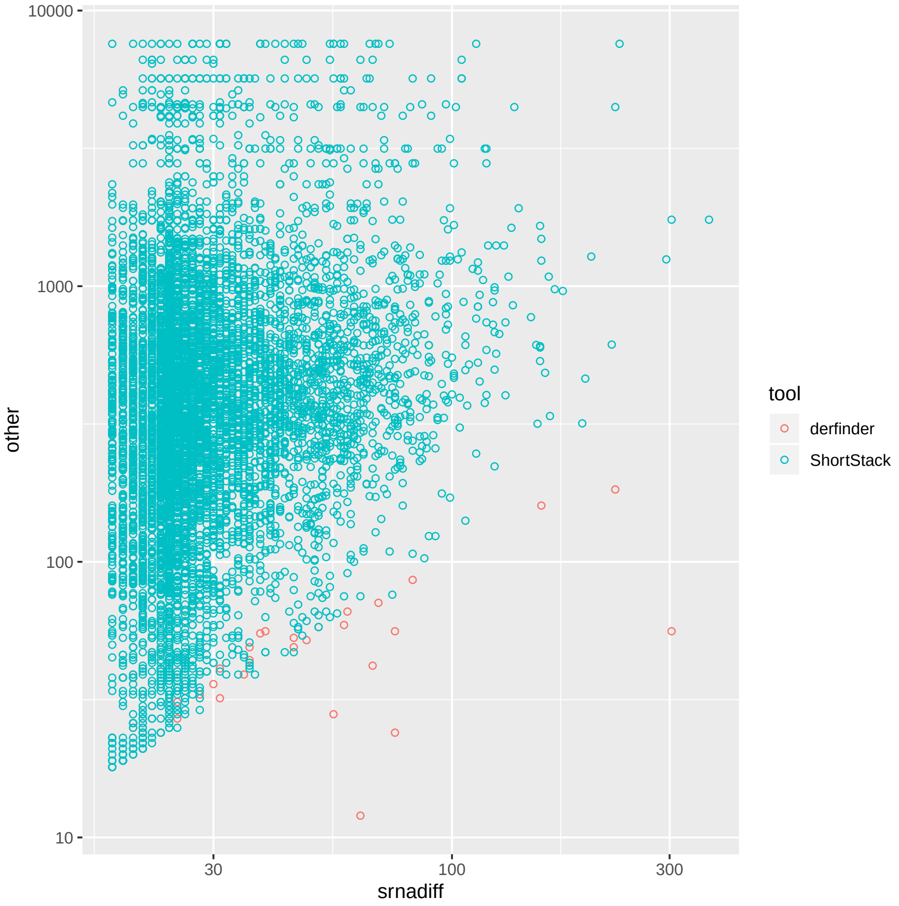

Figure 8: **Pairwise region size distribution.**

Number of features per region

Figure 9 confirms that predicted regions overlap at most one annotation.

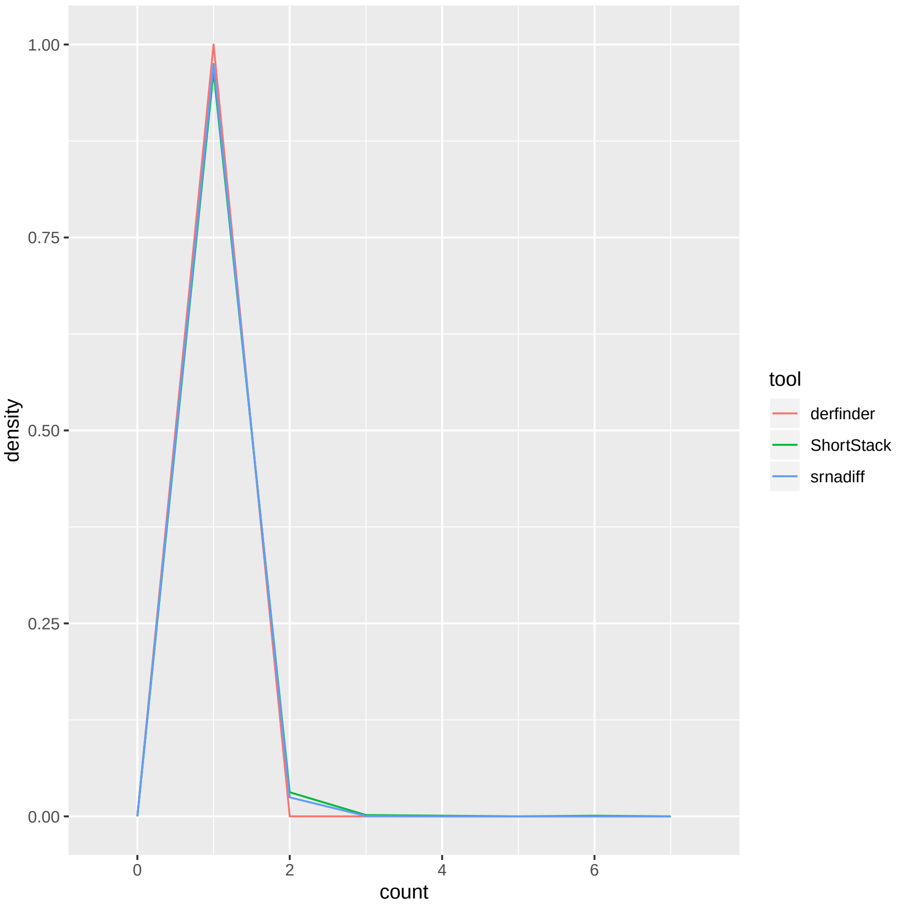

Figure 9: **Number of overlapped annotation per region.**

Pairwise p-value comparisons

Figure 5 confirms that derfinder finds higher p-values.

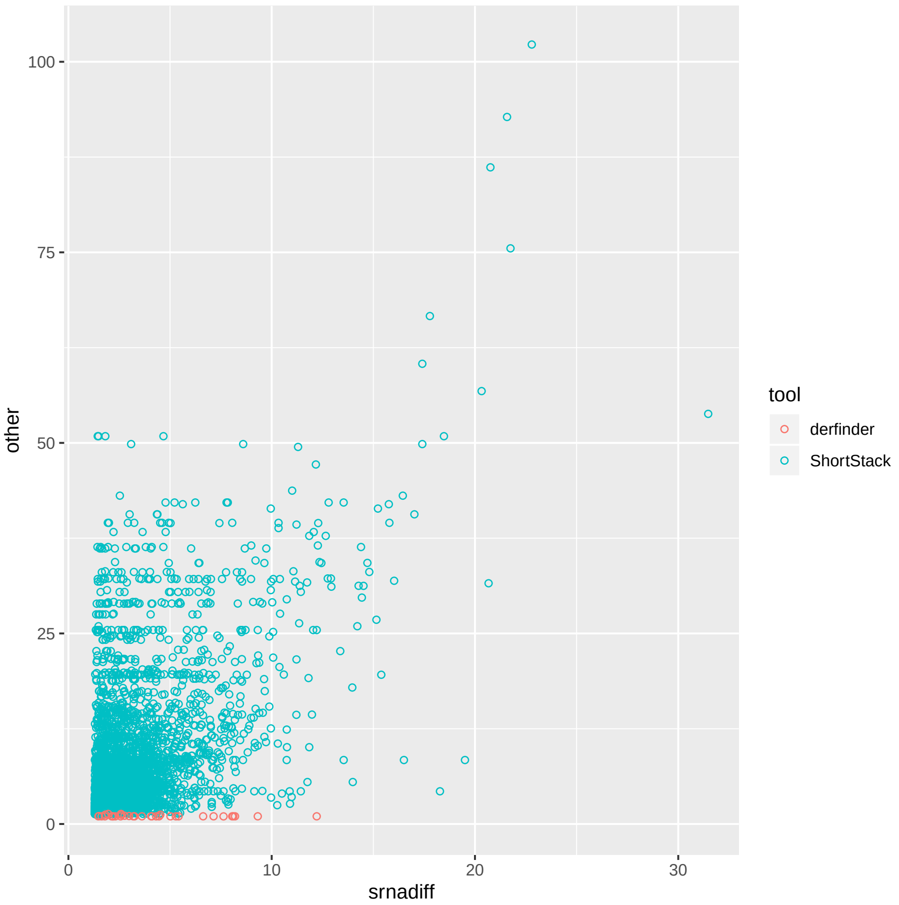

Figure 10: **Pairwise adjusted p-value comparisons.** The distribution of the $-\log_{10}$ of these values are plotted.

D. melanogaster dataset

The *D. melanogaster* datasets has a similar profile as the previous one. Results are given in Figures 11, 12, 13, and 14.

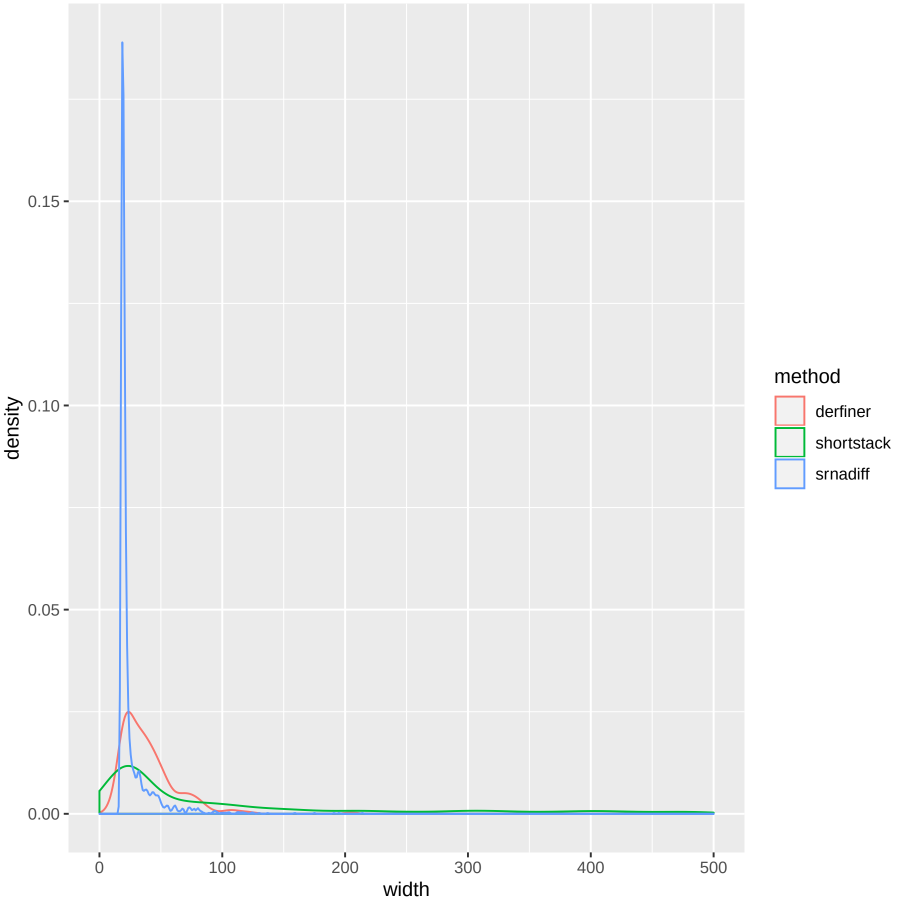

Figure 11: **Region size distributions.**

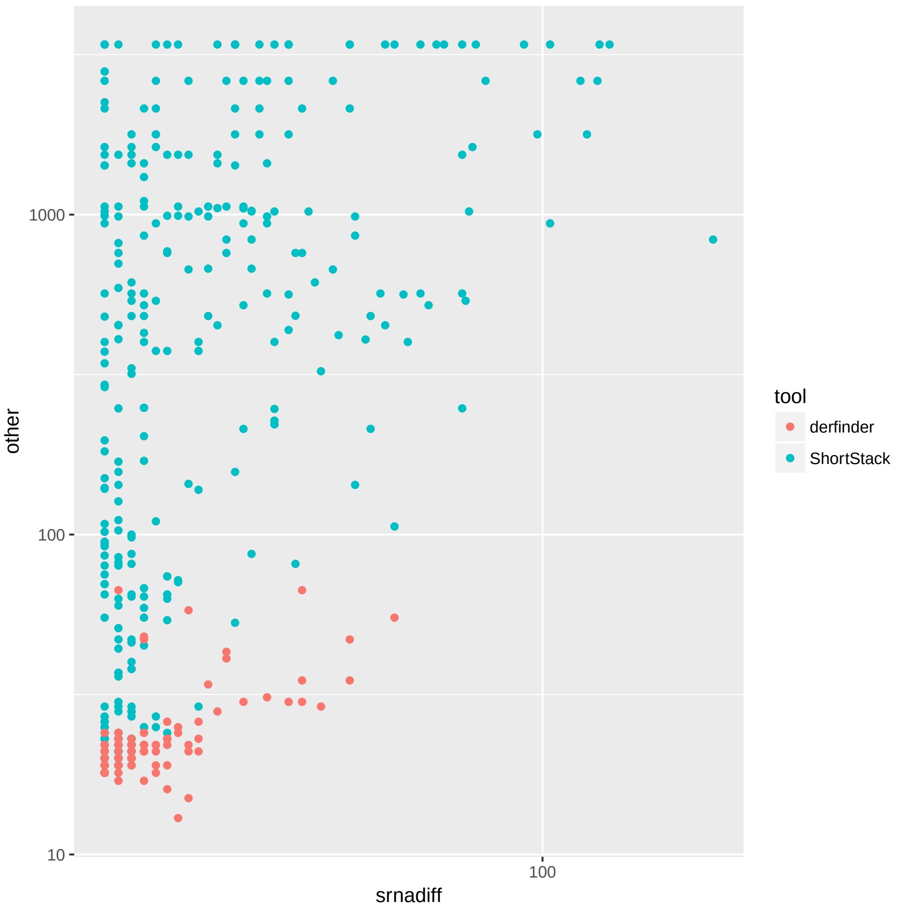

Figure 12: **Pairwise region size distribution.**

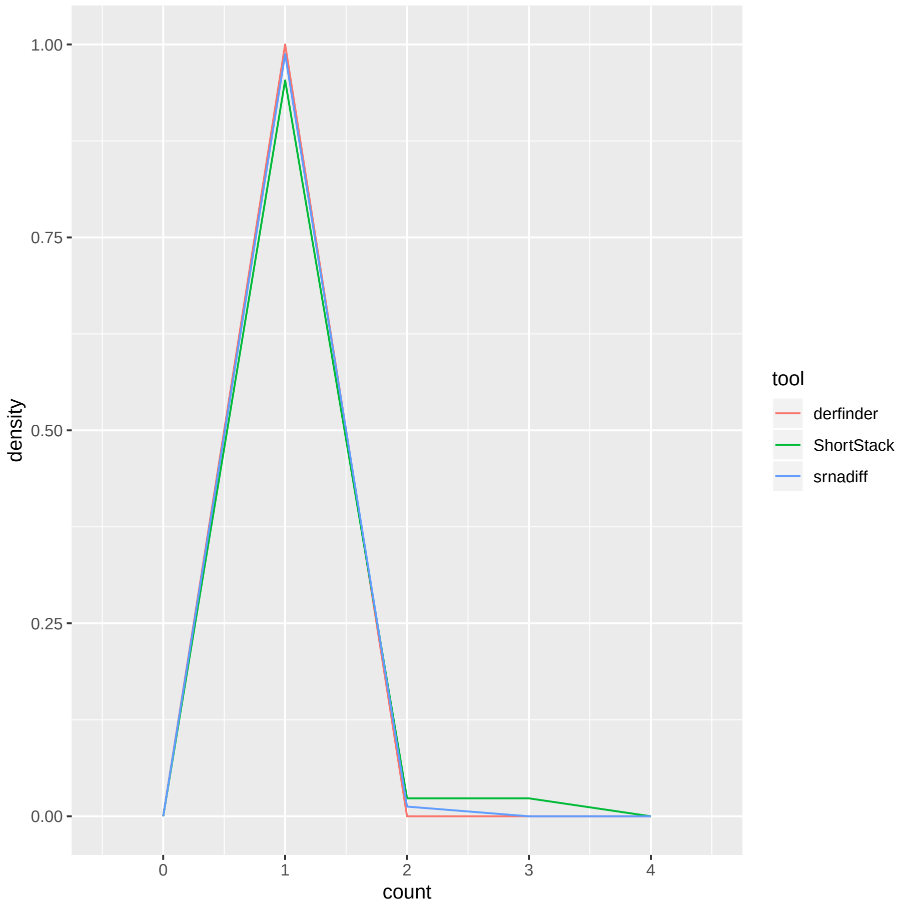

Figure 13: **Number of overlapped annotation per region.**

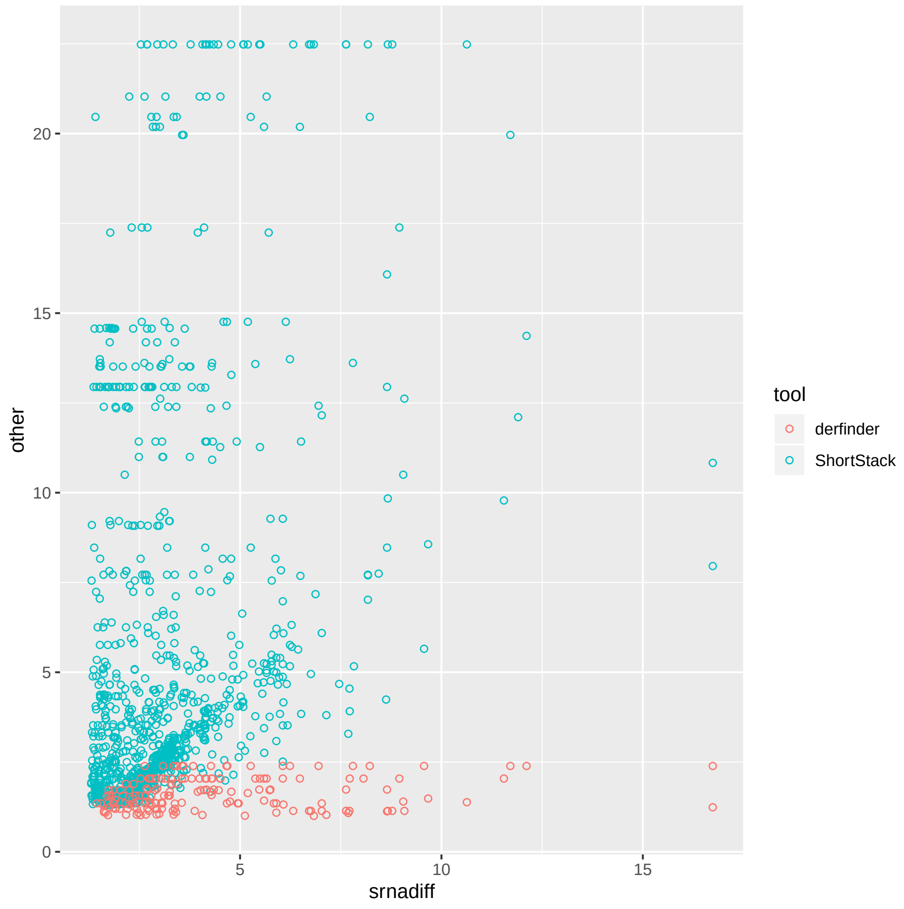

Figure 14: **Pairwise adjusted p-value comparisons.**

Simulated dataset

In this data set, derfinder does not find any good region. We thus only compare srnadiff with ShortStack.

As previously seen, ShortStack detects much larger regions (see Figure 15).

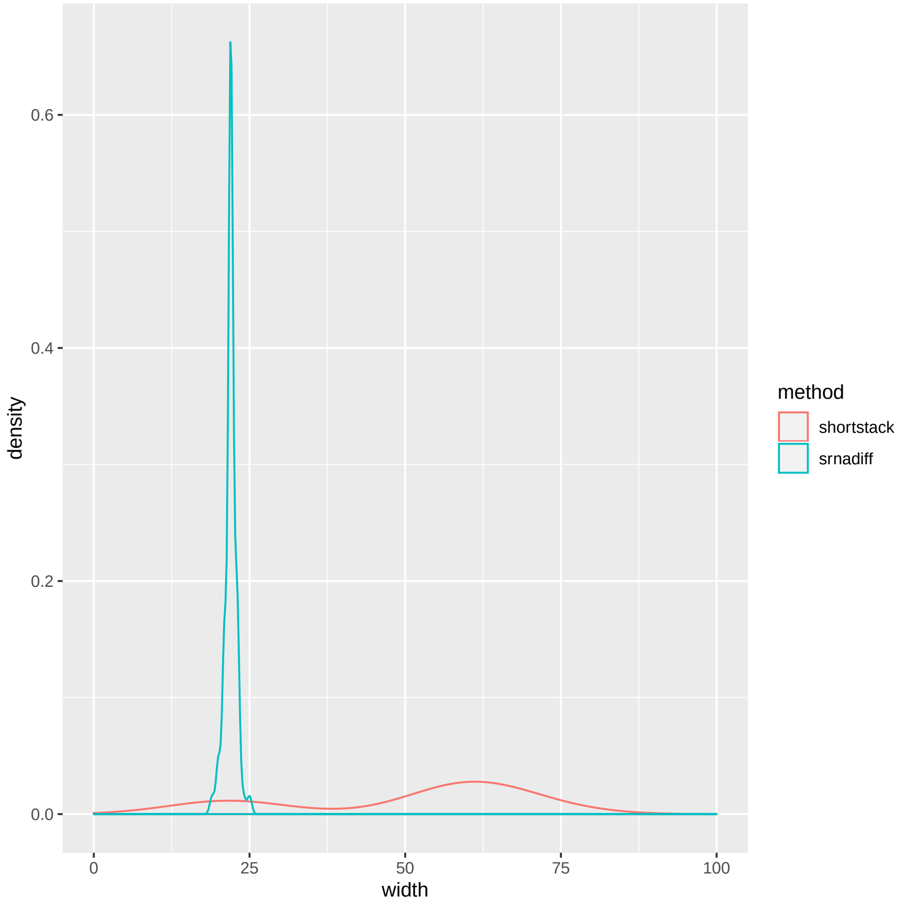

Figure 15: **Region size distributions.**

Figure 16 confirms that some differentially expressed regions found by ShortStack are longer, even when they co-localize with the regions found by srnadiff.

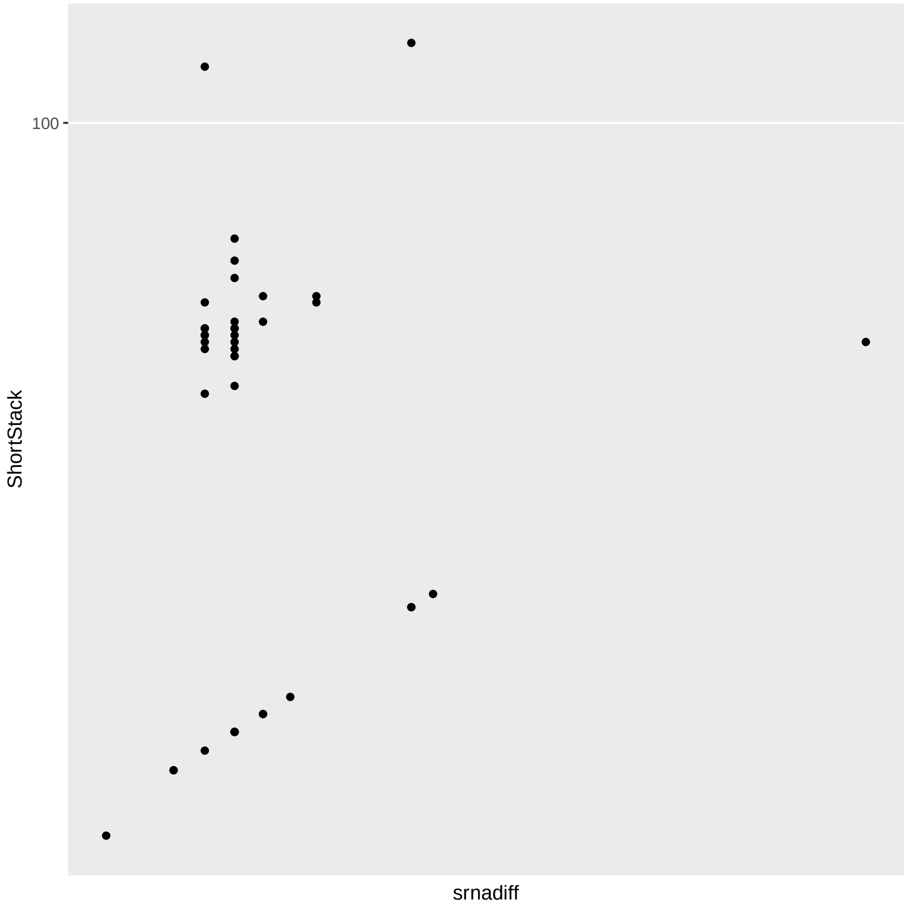

Figure 16: **Pairwise region size distribution.**

Figure 17 simply confirms that at most one annotation per detected region was found.

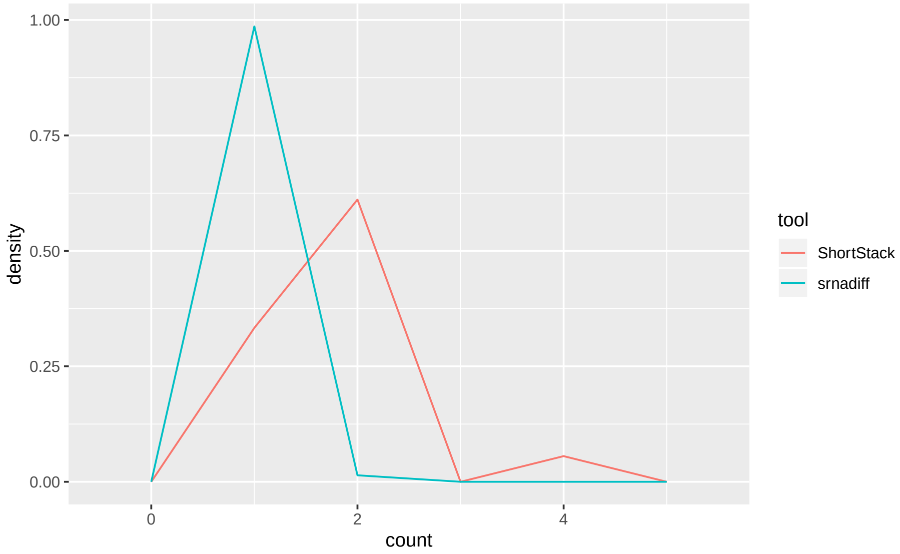

Figure 17: **Number of overlapped annotation per region.**

Figure 18 confirms that similar region give similar p-values.

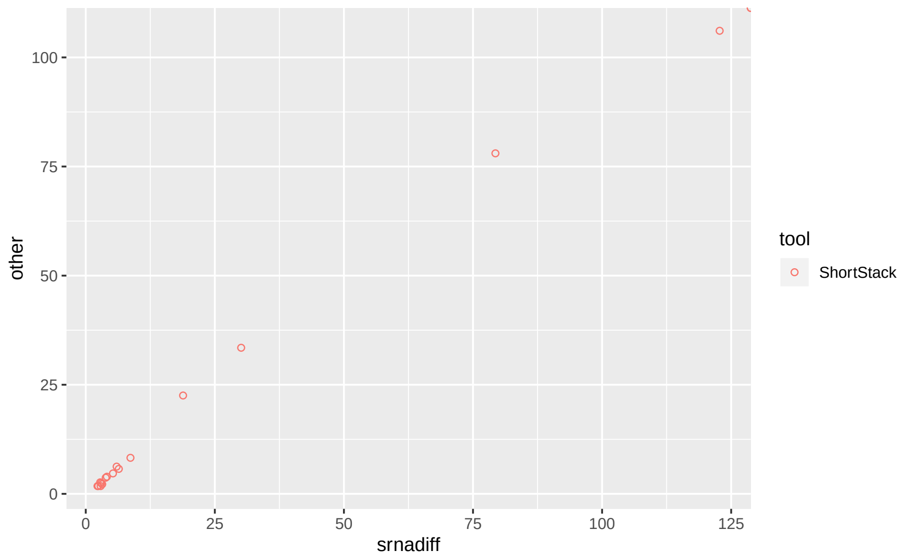

Figure 18: **Pairwise adjusted p-value comparisons.** The distribution of the $-\log_{10}$ of these values are plotted.

Commands used

Generating reads

We first extracted a set of miRNAs and piRNAs genomic intervals, stored in a GTF file called `annotation.gtf`. The script to generate the reads is:

```
nLines                  <- length(readLines(file("annotation.gtf")))
nonSelected             <- seq(nLines)
upReg                   <- sample(nonSelected, 100)
nonSelected             <- nonSelected[-upReg]
downReg                 <- sample(nonSelected, 100)
fold_changes            <- matrix(1, nrow = minLines, ncol = 2)
fold_changes[upReg, 1]   <- 2
fold_changes[downReg, 2] <- 2
simulate_experiment(gtf="annotation.gtf", seqpath="genome",
  reads_per_transcript=rpldis(nLines, 1, 1.5), num_reps=c(6, 6),
  fold_changes=fold_changes, outdir="output",
  paired=FALSE, readlen=30)
```

Pre-processing

Here are the first steps of the analyses:

- Trimming: `fastx_clipper -a` *adapter* `-l 15 -i` *reads.fastq* `-o` *reads_trim.fastq*
- Mapping: `bowtie -p 8 -m 1 --best --strata --chunkmbs 200 -S` *genome* *reads_trim.fastq* *reads.sam*
- Conversion to BAM, indexing: `samtools view -bS` *reads.sam* `| samtools sort -o` *reads.bam* `-`
- Indexing: `samtools index` *reads.bam*

Pre-processed data

All the pre-processed data, including the BAM files, the experimental design files, and the validated simulated differentially expressed regions, can be retrieved from `https://doi.org/10.15454/0DCIGO`

Quantification and test for differential expression

Here, we suppose that the annotation is stored in SAF format, a tabular format described by the authors of featureCounts, where the columns are: gene ID, chromosome, start position, end position, strand.

The `data` variable is the usual table of sample information (as used by DESeq2).

The R code for the quantification and test for differential expression follows:

```
library(DESeq2)
library(Rsubread)
counts <- featureCounts(data$FileName,
                        annot.ext="annotation.saf",
                        fracOverlap=0.5)
dds    <- DESeqDataSetFromMatrix(countData=counts$counts,
                                 colData=data,
                                 design=~Condition)
dds    <- DESeq(dds)
res    <- results(dds, 0.05)
```

srnadiff

Here is the code for srnadiff. `data` is the same table of sample information.

```
library(IRanges)
library(GenomicRanges)
library(srnadiff)
exp     <- sRNADiffExp(NULL, data$FileName, data$SampleName,
                       factor(data$Condition))
exp     <- setNThreads(exp, 6)
exp     <- runAll(exp)
diffReg <- regions(exp, 0.05)
writeLines(paste(seqnames(diffReg), start(diffReg),
                 end(diffReg), sep = "\t"), "output.bed")
```

derfinder

derfinder proceeds chromosome after chromosome. It takes BigWig files as input, instead of BAM files. The variable `chrs` stores the list of chromosomes.

```
library(derfinder)
files <- rawFiles(datadir=dir, samplepatt='.bw$',
                  fileterm=NULL)
names(files) <- gsub('.bw', '', names(files))
fullCov <- fullCoverage(files = bamFiles, chrs = chrs)
collapsedFull <- collapseFullCoverage(fullCov)
sampleDepths <- sampleDepth(collapsedFull, probs = 1)
rm(collapsedFull)
models <- makeModels(sampleDepths,
                     testvars = data$Condition)
cutoff <- 5
nP <- 10
derfinderReg <- list()
for (chr in chrs) {
  chrCvg <- loadCoverage(files = bamFiles,
```

```
                            chr = chr,
                            cutoff = cutoff)
  results <- analyzeChr(chr = chr,
                          coverageInfo = chrCvg,
                          models = models,
                          cutoffFstat = 5,
                          cutoffType = "manual",
                          nPermute = nP,
                          groupInfo = data$Condition,
                          writeOutput = FALSE,
                          returnOutput = TRUE,
                          runAnnotation = FALSE,
                          seeds = 140923 + seq_len(nP),
                          chrsStyle = NULL)
  derfinderReg[[chr]] <- results$regions$regions
}
derfinderRes <- unlist(as(derfinderReg, "GRangesList"))
```

ShortStack

We merged all the mapped reads into a unique BAM file, and run ShortStack with the following method.

`ShortStack --bamfile` *reads.bam* `--genomefile` *reference.fa* `--nohp --outdir` *output*

The quantification and test is then done as previous described.

Effect of some parameters

We wanted to test the effect of some parameters on srnadiff. srnadiff contains about 20 parameters, and a thorough test is out of the scope of the article. We chose the four most significant parameters, and assign them to different values along a large scale. The results are compared in terms of precision, recall, and F_1 score (presented in the main document).

Notice that srnadiff can give slightly different results with the same input, because when two putative regions are almost similar and have the same sizes, one of them (chosen arbitrarily) is discarded.

Moreover, different test beds will deliver somewhat different results.

Minimum coverage

The minimum coverage is the minimum number of reads per nucleotide to consider that a given region is expressed. The default is 10. Table 1 shows that these parameter, on this dataset, is optimal when set to 3. In our experiments, this parameter heavily depends on the dataset.

Table 1: **Results when changing the minimum coverage.**

threshold	# regions	TP	precision	recall	F_1
1	49	44	90%	100%	95%
3	70	44	63%	100%	77%
5	74	44	60%	100%	75%
10	71	43	61%	98%	75%
20	44	35	80%	81%	80%
50	32	27	84%	61%	71%
100	23	19	82%	43%	57%
500	10	8	80%	18%	30%
1000	8	5	63%	11%	19%

Merge distance

In the naïve method, two clusters are merged if they are distant by no more than d nucleotides, d being the merge distance parameter. The default is 100. Table 2 shows that these parameter, on this dataset, is optimal when set to 10, but results are quite stable along the the range of possible values.

Table 2: **Results when changing the merge distance.**

distance	# regions	TP	precision	recall	F_1
1	49	44	90%	100%	95%
10	71	43	61%	98%	75%
100	23	19	83%	43%	57%
1000	8	5	63%	11%	19%

Emission threshold

The HMM contains two states, each one following a Bernoulli distribution. The state "not differentially expressed" takes a value in $(t, 1]$ with probability p_1, and the state "differentially expressed" take a value in $[0, t]$ with probability p_2. Here, we modify the threshold t.

The default is 0.1, which is not the best value in this dataset (see Table 3), but the results are also quite stable.

Table 3: **Results when changing the emission threshold.**

threshold	# regions	TP	precision	recall	F_1
0.1	71	43	61%	98%	75%
0.3	47	43	91%	98%	95%
0.5	44	43	98%	98%	98%
0.7	43	42	98%	95%	97%
0.9	42	41	98%	93%	95%

Transition probability

We modified the probability from the "not differentially expressed" state to the "differentially expressed" state (default: 10^{-3}), and from the "differentially expressed" state to the "not differentially expressed" state (default: 10^{-6}) The results do not change at all when this value is changed.

Scalability

We generated synthetic datasets of different sizes to assess the observed time and space complexities of srnadiff. To do so, we generated the biggest dataset (also using polyester) that did not trigger a memory error on our machine. Then, we randomly sub-sampled the reads, and proceeded as previously. Time complexity is given in Table 4. The space complexity do not change significantly, as it bounded by the annotation size, and not the reads file size. Since they are simulated data, we could also compute a set of reference differentially expressed genes, that we took as truth standard (here, we found 50 such regions), based on the largest dataset. The table also provided precision, recall, and F_1 score for each dataset.

Table 4: **Time, memory usage, and classification statistics of different datasets.** Time is given in seconds. The number of reads is the average number of reads per sample (k: in thousands, M: in millions).

# reads	250k	3M	6M	30M	50M
time complexity	17	57	105	505	1386
# regions	78	71	71	71	79
TP	42	42	42	42	43
precision	54%	60%	60%	60%	54%
recall	95%	95%	95%	95%	98%
F_1	69%	73%	73%	73%	70%

Time increases roughly linearly with the number of reads.

Even though the sequencing depth is as low as 250 thousands reads, there does not seem to have a clear correlation between sequencing depth and precision nor recall.

Preprocessing time and memory usage

Time and memory usage of the deepTools command to generate the bigWig is given in the first columns of Table 5. The computation was done with 6 cores. Notice that the computation, done in Python, can take up to 4GB, and take more than 2 CPU-hours.

Time and memory usage of the samtools command to merge the BAM files is given in the last columns of the same table. They are negligible when compared to the other computations.

Table 5: **Time and memory usage of the preprocessing.** The second and third columns describe the BAM to bigWig conversion, and the last two columns the BAM file merging. In each cell, the first number is the time per CPU (in seconds), and the second is the RAM required (in MB).

method	BAM to bigWig		Merge BAM	
H. sapiens	29,218	2947	996	12
A. thaliana	494	440	136	11
D. melanogaster	1508	490	151	13
Simulated	31,135	4487	252	12

Versions of the tools used

- bowtie: 7.3.0
- deeptools: 3.1.1
- fastx_clipper: 0.0.14
- R: 3.6.1
- derfinder: 1.18.3
- DESeq2: 1.24.1
- polyester: 1.20.0
- Rsubread (for featureCounts): 1.34.6
- samtools: 1.9
- ShortStack: 3.8.5
- srnadiff: 1.5.2
