# Supplementary material for: Finding differentially expressed sRNA-Seq regions with srnadiff
Source: PLoS One. 2021 Aug 20;16(8):e0256196. doi: 10.1371/journal.pone.0256196 (PMC8378736; doi:10.1371/journal.pone.0256196)
Supplement: Attachment — Submitted filename: rebuttal.pdf [file pone.0256196.s003.pdf]

Dear Editor,

First, we thank you very much for this thorough review.
We tried to answer all the suggestions and comments addressed by the reviewers, in the paper, as well as in the package.

Please find hereafter our detailed answers.

Best regards,

Ignacio Gonzalez and Matthias Zytnicki.

=====================

Please use the space provided to explain your answers to the questions above. You may also include additional comments for the author, including concerns about dual publication, research ethics, or publication ethics. (Please upload your review as an attachment if it exceeds 20,000 characters)

Reviewer #2: (No Response)

Reviewer #4: The manuscript by Zytnicki and Gonzales describes srnadiff, a new computational method for calling differentially expressed small RNAs de novo, without relying on genome annotation. This direction is biologically attractive, especially given a variety of cell type-specific small RNAs that can be missed in a basic annotation, including, but not limited to, new miRNAs, piRNAs, tRNA fragments, and possibly enhancer RNAs (eRNAs). The implementation of this method is based on DESeq–based analysis of differential expression supplemented by a few straightforward computational steps. The description of the method itself is mostly sufficient, although I have some questions about the choices of methodology. The description of results produced by this method and their evaluation needs more serious attention. For a potential user, it would be important to know what new additional information this method can provide as compared to both the basic method based on pre-existing annotation and to other comparable methods.

Major comments:
1. It looks like positional resolution of this method is 1 bp as suggested by the use of run-length encoding. The standard statistical null model of DESeq, however, is optimized towards the analysis of read counts over whole transcripts. The CPMs/read counts calculated at 1-bp resolution across the genome most likely follow a different random statistical distribution compared to the set of full-length transcripts. Was this assessed?
==> We used DESeq2 twice in the pipe-line. First, as a preprocessing step of the HMM method, and second, in order to give a p-value of the candidates in the last step.
In the latter context, we do not use the run-length encoding version, but we do in the former context. As you commented, it is true that DESeq2 has not been designed for that, and we did not provide the reader enough material to make sure that this use was in line with DESeq2 statistical assumptions.
We added this in the article:
===
This use of DESeq2 is obviously not standard, because DESeq2 expects the total number of reads per transcript, and not the nucleotide-wise number of reads (also called \emph{coverage}).
However, we can first notice here is that, in an ideal case of a miRNA, piRNA, or snoRNA, where all the reads do map at the very same positions and cover the full transcript, the total number of reads and the coverage are exactly identical.
We also plotted several quality control distribution, and checked that they behave as expected.
First, we plotted the dispersion estimation.
Briefly, the read counts for a particular gene are expected to follow a negative binomial distribution, with two parameters: the mean, and the dispersion.
The dispersion is estimated using the mean, and is expected to follow a trend for the majority of the genes.
If many genes depart from the trend, the expression is clearly poorly estimated.
In Supplementary Figure 1a, we show that, on the human dataset, the dispersion plot is close to the expected one.

This confirms that the estimation step was correctly carried out.
Second, we plotted the p-value distribution.
The p-value is expected to be uniform under the null hypothesis, and close to 0 under the alternative hypothesis.
The supplementary figure 1b confirms that we observe a mixture of the two distributions on the human dataset.
===

2. Line 121-122:
If the method's performance does not depend on the values of HMM parameters, is HMM the optimal computational framework to use?
This lack of dependency may suggest a problem with the assumption of the process based on nucleotide-to-nucleotide transition, a problem with data sensitivity (low depth) etc.
==> Very true, although one could argue that HMM is robust in this context, which is probably much desired.

Of note, the HMM method does a good job at finding moderately, differentially expressed small RNAs: our results show it.

The main advantage with HMM is that it comes with the theoretical background (we exactly know what we optimize), and algorithms.

That said, HMM is maybe not adapted to very long, little expressed differentially expressed small RNAs.

This is why we complement it with two other methods.

IR, for instance, is better when finding long RNAs: among the 10 longest candidates for the human dataset, 9 are provided by the IR method.

We added this in the text:
===
Ideally, each method is made to complement each other.
The reason is that the expression profile of the small RNAs are very diverse: miRNAs are expected to pile up in short stacks, whereas piRNAs spread over thousand of nucleotides.
We specifically chose the HMM method, because it can detect short, moderately differentially expressed RNAs.
In turn, the IR method can detect longer patterns.
===

3. In the evaluation section, precision and recall are shown only as barplots. Would it make sense to present curves based on the ranked lists of predictions?
==> True. We changed barplots to ROC curves in Figure 5. We also changed the description accordingly.
===
The results of each tool as a receiver operating characteristic (ROC) curve, given in Figure 5.
The ROC curve provides the sensitivity against (1 $-$ specificity).
The area under the curve (AUC) gives the probability that a tool ranks a randomly chosen true candidate higher than a randomly chosen wrong one.
The figure shows that srnadiff clearly outperforms other tools.
===

4. Using previously annotated sRNAs as gold standard ("truth set") may be appropriate as a first-level evaluation, but it is not clear how much srnadiff adds to the basic analysis of pre-annotated sRNAs.
It would be important to address the predictions of differentially expressed sRNAs outside of basic annotations, overlaps of these predictions between the three tested methods; and to discuss possible biological relevance and examples of these predictions: for example, potential new miRNAs or piRNAs (hopefully confirmed by the presence of typical sequence patterns), tRNA fragments etc.
==> We added a new section in order to answer this question, and the following one.

Briefly, we added new databases (described in the Methods section), and tried to discover new miRNAs (using miRDeep2) and piRNAs (considering region size and distribution of the reads).

We reduced the number of unannotated differentially expressed regions to 101.

5. Lines 292-294: For a potential user, it would be important to know more about the new regions predicted

by srnadiff but not by the ShortStack method. How many of these regions are previously annotated and how many are novel? How these novel regions are distributed between the known functional genomic elements according to a standard annotation of the genome: introns, exons, enhancers etc? What is the length distribution of these novel regions?
It would also be important to show examples of newly predicted regions of potential biological interest, similar to the previous comment.
==> The new section mentioned in the previous paragraph also addresses this point.
Briefly, we added a plot showing genomic locations and another on showing the region sizes of the differentially expressed regions.
We organized the plots, so that the contribution of each tool is visible.
Finally, we added in Supporting Information two screenshots displaying differentially expressed regions, found by srnadiff and no other tool.

Minor comments:
1. Although the goal is to precisely define sRNA positioning in the genome, the 1 bp resolution of read counts may result in lower read counts and lower sensitivity of differential calls by DESeq.
==> This is true for messenger RNA sequencing but not exactly for sRNA. We added the following paragraph in the text.
===
This method is expected to give the same results as the annotate-then-identify method for all the sRNAs with a very sharp profile, such as miRNAs and tRFs.
Contrary to messenger RNAs, the reads spans the whole transcript.
Supposing that a sRNA, such as a mature miRNA, is sequenced x times, the annotate-then-identify will quantify an expression of x for this feature.
Our method will also find the same expression of x, because each nucleotide has a sequencing depth of x.
The only difference is that we will likely have identical counts for each nucleotide of the miRNA.
This is why, in our implementation, we collapse lines of the count matrix that have exactly the same counts into a unique row, because the counts actually describe the same object.
===

In a similar vein, what is a reasonable threshold of p-value (line 126) at which a genomic position is omitted from statistical consideration? Was it estimated in a rigorous way based on real data?
==> In the line you mention, there is no threshold of p-value.
Actually, the HMM needs a p-value for each nucleotide of the genome, and even a non significant p-value of 0.9 can be correctly interpreted by the HMM.
However, we did use a threshold for the coverage: by default, there should be at least one sample with at least 10 reads.
The aim here was to save computation time, with (almost) no decrease of sensibility.
We used this (admittedly arbitrary) threshold based on our experience.
A region which is covered by less than 10 reads, even in the most expressed sample, is not likely to be reliably called differentially expressed.
This parameters can be easily changed by the user, though.

2. English needs minor corrections and stylistic editing.
==> Thanks! We attentively read the manuscript, and tried to correct all the mistakes.

Reviewer #5: The work proposed a method called “srnadiff” in order to find differentially expressed sRNA, without annotation (the annotation is optional).

The authors divide the proposed approach into two steps. The first step comprises applying some methods like HMM (hidden Markov model), IR (Irreducible regions), annotation (as optional), etc. in order to produce genomic intervals that are potential differentially expressed regions.

The second step comprises clustering the samples and applying the DESeq2 method for the identification of differential expression.

Essentially, the problem addressed by the paper is not one of detecting differential expression, in numerical or statistical terms, but rather one of mapping, i.e., the contribution of the work is essentially in step 1.

Therefore, the manuscript presents an R package for the detection of differentially expressed smallRNAs. The text is difficult to understand and does not present clearly with tables and figures (see specific comments below). The authors do not sustain the reasons their results are better (identifying more differentially expressed sequences does not exactly mean that the tool is better), perhaps this difficulty in making clear is because of the way the comparison was done.

The work is interesting and applied in a significant bioinformatics research context. Some points could be better presented which could improve the quality of the work.

Major:

Regarding the presentation of the method, it would be very important to improve the presentation of step 1 by clearly contextualizing how the different adopted approaches are integrated to perform the clustering of genomic regions. Making clear the adopted criteria, information flow and parameters considered, among other information.
==> First, we reorganized the manuscript to improve the flow.
The description of the segmentation methods are now described in the "Step 1" section.
We also added some contextualization: for instance, before the description of Step 1 and 2, we added:
===
Briefly, the first step includes several, independent methods that detect putative differentially expressed regions.
The second step considers all the regions found by the previous step, and chooses the best ones.
===
We also added inputs and outputs, and parameters used, for each method.
Figure 1 has also been modified to include parameters, and a little cartoon of the data which are produced by each tool (coverages and intervals).

Explain and include references to which methods and implementations were adopted for the HMM and IR.
==> We did not make it clear that we implemented the method from scratch.
We added "The algorithm has been implemented from scratch in C++." in the corresponding paragraphs, in the "Strategy" section, and we cited again the reference of the IR method.
Concerning the Viterbi algorithm, being a textbook algorithm, there is no clear reference to cite.

The acronym snoRNAs is used but not previously presented as piRNAs for example.
==> Thanks, we corrected that in the introduction.

The method describes expression identification with only RNA-Seq data without reference, but in Figure 1 the first step uses a BAM (output file mapping reads to a reference).
==> This is a major misunderstanding, and we are sorry that we did not make it clear.
The method does not need a reference annotation (but can use it, if available), but does need a reference genome.
We tried to correct this, especially in the Abstract, where we mentioned "reference sequences of sRNAs".
The new text is now:
===
To the extent of our knowledge, srnadiff is the first tool that detects differentially expressed sRNAs without the use of external information, such as genomic annotation or additional sequences of sRNAs.
===
Moreover, we added to following lines in the introduction.
===
In this work, we will suppose that a reference genome is available.
Although many organisms are still not assembled, most model and many non-model organisms are available,

and the sequencing of the remaining organisms progress at a fast pace.
===

In step 2, the proposed work applies the DESeq2 method as a method for identifying differential expression. The proposed approach is available from a Bioconductor package. The suggestion to the authors is to make available to use it as a library and apply other methods for differential expression detection. An interesting proposal would be to apply the methods together, as proposed in this work:

https://journals.plos.org/plosone/article?id=10.1371/journal.pone.0190152
==> We usually think that, for a package, adding several tools is a bad practice.
First, it increases the number of dependencies, and makes a package more difficult to maintain.
Second, it confuses the user, because she/he does not know which tool is best.
We usually prefer benchmarking the tools, and finally choose the best one.
The example that you refer is a pipe-line, with different purpose in mind.
Yet, the reviewer is always right, so we added the tools.
We considered the four tools which were mentioned in the article, but discarded NOISeq, because it does not seems maintained any longer.
We added a new paragraph, called "Computation of the p-values" in the text.
The text is:
===
p-values at computed twice in the process.
First, they are computed for each position of the genome in the HMM strategy.
Second, candidate regions are tested for differential expression, and given a p-value.
By default, DESeq2 is used to perform the two steps.
However, for the sake of completeness, we made two other widely used packages available for the user: edgeR, and baySeq.
The alternative package can be chosen by specifying the parameter diffMethod in the srnadiff function.
===
We also added Figure 8, which compares the 3 packages on the simulated dataset, and shows that the DESeq2 (default) method gives best results.

The tool itself is well done and seems to me to be really necessary, even for computational efficiency, but the way it is presented can be much improved, despite the corrections already stated in revision 1.

Regarding the results, in the presentation in Fig. some works are mentioned (article 1, article 2, usual differential expression calling method). It is important to contextualize these works in the text, justify the choice of these papers and their characteristics so that it is possible to compare them contextually with the proposed approach.
==> We are sure about the Figure you mentioned.
Anyway, we now explicitly present articles 1 and 2 as reviews on plants and animals on small RNAs.
===
These sRNAs include the well-known microRNAs, but also tRNA-derived RNA fragments (tRFs), small interfering RNAs (siRNAs), Piwi-associated RNAs (piRNAs), small nucleolar RNAs (snoRNAs), which have been extensively studied in plants and animals (see [1, 2] for reviews on sRNAs on both kingdoms).
===
In the "Benchmark" section, we specified that we chose three random studies, so that they represent the diversity of the small RNA approaches.
===
For the published datasets, we randomly choose several ones from the vast literature, with few criteria.
First, there should be replicates, second the differentially expressed elements should be accessible as supplementary data of the article.
We then wanted to encompass a variety of model organisms: Homo sapiens, Arabidopsis thaliana and Drosophila melanogaster.
The analysis used by the three articles are also quite different, as well as the sequencing machines.
===

Moreover, for each dataset, we present the method which has been used by in the original work.

For the human dataset:

====

In the first paper, the authors mapped the reads with bowtie on miRBase.

The miRNAs were tested for differential expressed with edgeR.

In the second paper, the authors mapped the reads with Novoalign on the reference genome.

They downloaded piRNA and snoRNA annotations, and quantified their expression with the BedTools.

They were tested for differential expressed with edgeR.

====

For the thale cress dataset:

====

The authors mapped the reads with RazerS on the reference genome.

Reads co-localizing with known miRNAs were kept for further analysis.

The authors then looked for other, unknown, putative miRNAs.

Other reads were compared to known cDNAs, miRNAs, tRNAs, rRNAs, snoRNAs, and other RNA sequences, and matching reads were excluded.

Remaining reads were then given to miRDeep and miRCat, two miRNA finding tools.

The new set was added to the previous, known set of miRNAs.

Differential expression analysis was performed with a X2 test.

====

For the fruit fly dataset:

====

The authors mapped the reads with bowtie on the reference genome.

Detection of new miRNAs, and quantification of known and new miRNAs, was performed with miRDeep2, using data from miRBase.

Micro RNAs were tested for differential expressed with edgeR.

====

Fig. 4 can be better presented and contextualized. It is important that the authors make clear the numbers and definitions of what they considered as True Positive and True Negative for each of the datasets. In addition, it is important that they present the Precision (TP/(TP+FP)) and Recall (TP/(TP+FN)) rates in order to make clear the improvement provided by the proposed method. Clarify that the method recovers regions with differential expression and with precision, i.e., without an excessive number of false positives. Also, correct the y-axis labeling of the figures. It would interest to make clear what these results show. It is important to explain and contextualize where this better result can be visualized and why it is described as "better".

==> Thank you for the advice, we tried to follow it.

We clarified the context, and we focused on recall, but no precision, because we have no exhaustive true set (and it is very complicated to obtain it).

However, we mentioned that a precision will be assessed in the simulated dataset.

Of note, we did not mention that a tool was "better", but that some tools (such as srnadiff) missed less putative regions.

We explained this in the "Benchmarking" section.

===

Of note, it is not possible to assess the number of false positives, \textit{i.e.} the regions detected by the evaluated tools, on real-life datasets, because there exists no method that finds all the differentially expressed small RNAs.

Therefore, metrics such as specificity or precision cannot be collected on these dataset.

Our aim, here, is to test whether the evaluated tools are able to detect all the regions that have been classified as differentially expressed by the reference methods.

The simulated dataset, even though it is imperfect because it poorly reproduces the erratic distribution of the reads observed in real-life datasets, can assess both the number of false positives, and false negatives.

This is why we added a fourth, synthetic dataset, to the real-life ones.

===

Before Figure 4, we clarified what we called "true positive".

===

Figure 4 shows the recall of the different methods.

The recall (or sensitivity) is defined as TP/(TP+FN), where TP is the number of true positives, and FN is the number of false negatives.
In our benchmark, a true positive is region which has been detected as differentially expressed by a reference set (here, the miRNAs published in the articles), such that at least 80\% of the nucleotides overlap with a region detected as differentially expressed by srnadiff (or other evaluated tools such as derfinder, and ShortStack).
Conversely, the false negatives are the regions which has been detected as differentially expressed by a reference set, which do not overlap (with at least 80% of the nucleotides) with a region detected as differentially expressed by the evaluated tools.
===

Fig. 3 refers to the comparison tools as source, 4 tool, 5 and 6 method.
==> Yes! We used "tool" to be more consistent.

Fig. 6, Y-axis should be better identified what represents “value”?
==> Yes! We mentioned that the top plot was "time (in s)", and the bottom "space (in Mb)".

The p-value was defined for all methods, however the fold-change is mentioned only for synthetic dataset. It is important to make clear the adopted p-value and fold-change for all methods and datasets.
==> We already mentionned the p-value thresholds in the "Benchmark" section:
===
srnadiff was run with no annotation, and an adjusted p-value threshold of 5%.
We also run derfinder on the same datasets, with a q-value of 10%.
We used a third method, which first clusters the reads with ShortStack (comparing several clustering methods is out of the scope of this article), quantified the expression of the regions found by ShortStack with featureCounts, tested for differential expression with DESeq2, and kept the regions with an adjusted p-value of at most 5%.
[...]
The reason why we chose a q-value of 10% for derfinder, instead of 5%, is that the statistics produced by derfinder is significantly more conservative, and it produces much less predicted regions than other approaches.
For a fair comparison, we decided to lower the stringency for this tool.
===
We added that no fold change was applied.
Of note, srnadiff now provides the log2FC of the called regions.

The preprocessed dataset used to generate the results was not made available by the authors. It is recommended that the dataset used be made available that allows the replication of the results by the research community.
==> Very true.
We uploaded all the BAM files, the experimental design files, to a public dataverse with a persistent DOI: https://doi.org/10.15454/0DCIGO
This information has been added in Supporting Information.

Minor:

- Fig. 2 low resolution.
==> Fixed!
