# Supplementary material for: Finding differentially expressed sRNA-Seq regions with srnadiff
Source: PLoS One. 2021 Aug 20;16(8):e0256196. doi: 10.1371/journal.pone.0256196 (PMC8378736; doi:10.1371/journal.pone.0256196)
Supplement: Attachment — Submitted filename: rebuttal.pdf [file pone.0256196.s004.pdf]

Dear Reviewer,

Again, we thank you very much for the time you spent to improve our manuscript.

Please find hereafter the answers to your suggestions.

Best regards,

Ignacio González and Matthias Zytnicki.

My main current concern is the language, especially in the new edits.
==> We carefully read the text again, and corrected a few typos and errors.

These are just a few examples:
P. 4 – need clarifying:
'Ideally, each method is made to complement each other. The reason is that the expression profile of the small RNAs are very diverse'
==> We replaced the sentences with the following ones:
===
Indeed, expression profiles are very specific to each of the sRNA classes: miRNAs pile up in short stacks, whereas piRNAs spread over thousand of nucleotides.
In order to cope with this diversity, we found that two different methods yielded better results than a unique one.
The HMM method efficiently detects short, moderately differentially expressed RNAs.
The IR method can detect longer patterns.
===

P. 6: poor language:
'The algorithm has been implemented from scratch in C++.'
==> In the HMM section, we replaced the sentence with:
===
We implemented this Viterbi algorithm, optimized for our purposes, in C++.
===
==> In the IR section, we replaced the sentence with:
===
We implemented the algorithm in C++, using the description from [22].
===

P.8: a few passages need editing:
'Concerning the published datasets, we randomly choose them from the vast literature, with few criteria…
The analysis used by the three articles are also quite different, as well as the sequencing machines.'
==> We replaced the sentences with:
===
We selected several datasets meeting the following criteria.
First, the experimental design should include replicates in each condition.
Second, the differentially expressed elements found by the authors should be accessible as supplementary data of the article.
Third, we wanted to include a variety of model organisms: *Homo Sapiens*, *Arabidopsis thaliana* and *Drosophila melanogaster*.
Fourth, the sequencing machines should be different, in order to include the diversity of the machines in our benchmark.
Last, the analysis pipe-lines should also be different.
===

‘The simulated dataset, even though it is imperfect because it poorly reproduces the erratic distribution of the reads observed in real-life datasets, can assess both the number of false positives, and false negatives. This is why we added a fourth, synthetic dataset, to the real-life ones.’
==> We replaced the sentences, with the following, shorter, one, which fits better right after the previous paragraph:

===

In order to assess the number of false positives, we added a last, simulated, dataset.

===

P. 12: Unclear: does this mean that no novel piRNAs were detected by the new method?
‘This seems to indicate that no differentially expressed piRNA was missed.’
==> True, the sentence was unclear.

In the previous paragraph, we showed that, among the unannotated differentially expressed regions, 30 were in fact piRNAs (using an additional database).

In the paragraph mentioned by the reviewer, we showed that the remaining unannotated differentially expressed regions were short, with no bias U/A bias on the first/tenth position.

So, we changed the sentence to:

===

This seems to indicate that the unannotated differentially expressed regions are probably not piRNAs.

===

The new supplemental figure with genomic tracks of the examples of new detected small RNAs also needs additional work. As of now, it is just a screenshot from IGV, where it is difficult to read both vertical and horizontal scales, and there are many unnecessary image elements that should be removed.
==> The image has been modified, so that every information is easily readable.

Unnecessary information has been removed.
